# Prevalence and factors of elevated alanine aminotransferase in central Taiwan – a retrospective study

**DOI:** 10.7603/s40681-016-0011-7

**Published:** 2016-05-09

**Authors:** Hsien-Feng Lin, Shih-Wei Lai, Wen-Yuan Lin, Chiu-Shong Liu, Cheng-Chieh Lin, Ching-Mei Chang

**Affiliations:** 1 School of Chinese Medicine, China Medical University, 404 Taichung, Taiwan; 2 College of Medicine, China Medical University, 404 Taichung, Taiwan; 3 Department of Family Medicine, China Medical University Hospital, 404 Taichung, Taiwan; 4 Department of Nursing, Tungs’ Taichung Metro Habor Hospital, 435 Taichung, Taiwan

**Keywords:** ALT, Hepatitis B, Hepatitis C, Hypertriglyceridemia, Obesity

## Abstract

**Background:**

The aims of this study were to assess the prevalence of elevated alanine aminotransferase (ALT) and explore its related factors in Central Taiwan.

**Methods:**

The study employed a retrospective design. The study selected a sample of 5,550 subjects between the years 2000 to 2004. The indivduals undergoing health examinations in a medical center in Central Taiwan were enrolled as subjects for this research. The patients’ demographics, smoking and drinking habits, laboratory findings, and abdominal ultrasound results were collected and analyzed. Correlations between variables were analyzed using SPSS/ PC Windows for frequency distribution, *t*-test, *Chi*-square test, and multivariate logistic regression.

**Results:**

There were 3103 men (55.9%) and 2447 women (44.1%). The mean age was 49.4 ± 12.3 years (age range of 20-87). The overall prevalence of elevated ALT was 17.1%, with a significant gender difference (23.2% in men *vs.* 9.4% in women, *P* < .0001). The multivariate logistic regression analysis showed that the factors significantly related to elevated ALT were central obesity, hypertriglyceridemia, and anti-HCV positive in men and women.

**Conclusions:**

Central obesity, hypertriglyceridemia, and anti-HCV positive are factors predominantly related to elevated ALT in men and women.

## 1. Introduction

In 2003, chronic liver disease and cirrhosis were the sixth leading causes of death, and liver cancer was the leading cancer in Taiwan [1]. Thus, Taiwan is an area endemic for chronic liver disease [2]. Measuring alanine aminotransferase (ALT) is a very common test performed during routine screenings. Increasing evidence indicates that histologically advanced liver disease (*i.e.,* cirrhosis) may accompany normal or minimally elevated aminotransferase levels [3-5]. Further compounding this issue is the elevated ALT and AST are commonly detected in apparently healthy persons [6].

The prevalence and correlation of elevated aminotransferase have been documented in previous studies of certain population groups (*i.e.,* blood donors and obese adolescents) [7-9]. However, data on the prevalence of elevated aminotransferase in the general population is limited. To gain a better understanding of the burden of liver disease in Taiwan, we employed a retrospective research methodology to assess the prevalence of elevated ALT and explore factors related to it.

## 2. Materials and methods

This was a retrospective research study. From January 2000 to December 2004, we analyzed the medical records of all the people receiving self-referred health examinations at one medical center located at Taichung city in Taiwan. In Total, 5,550 subjects were included in our analysis. The ethics committee of the medical center approved this retrospective study. Complete physical examinations were performed by the doctors at the Department of Family Medicine.

We collected patients’ demographics, smoking and drinking habits, blood pressure, body mass index, waist circumference, laboratory findings, and abdominal ultrasound results for analysis. Abdominal sonography was performed by gastroenterologists using a high resolution real-time machine (TOSHIBA Sonolayer SSA-270A, convex-type 3.5 MHz transducer, Tochigi-Ken, Japan). Blood pressure was measured using a mercury sphygmomanometer with patients in a sitting position. Body mass index (BMI) was measured as follows: weight (kg) ÷ height (m)^2^. Waist circumference (WC) was taken midway between the inferior margin of the last rib and the crest of the ilium along a horizontal plane [10]. Generalized obesity was defined as BMI ≥ 27 [11]. Central obesity was defined as WC ≥ 90 cm for men and ≥ 80 cm for women [11]. Venous blood samples were obtained in the morning after a 12 h overnight fast. A number of biochemical markers, such as alanine aminotransferase (ALT), total cholesterol (TC), triglyceride (TG), low density lipoprotein cholesterol (LDL), high density lipoprotein cholesterol (HDL), fasting glucose and uric acid, were analyzed using a biochemical autoanalyser (Hitachi 736-15, Tokyo, Japan) at the Department of Clinical Laboratory of this medical center within 4 h of their collection. For our study, hypercholesterolemia was defined as total cholesterol ≥ 200 mg/dl [12]. Hypertriglyceridemia was defined as triglyceride ≥ 150 mg/dl [12]. Elevated LDL was defined as LDL ≥ 130 mg/ dl [12]. Reduced HDL was defined as HDL < 40 mg/dl for men and < 50 mg/dl for women, respectively [12]. Subjects were considered to have diabetes mellitus if fasting glucose was ≥ 126 mg/dl or subjects with a family history of diabetes [13]. Subjects were considered to have hypertension if the average blood pressure reading of both hands exceeded 140 mmHg systolically and/ or 90 mmHg diastolically or subjects had a family history of hypertension [14]. Hyperuricemia was defined as serum uric acid ≥ 7.0 mg/dl in men and ≥ 6.5 mg/dl in women [15]. Elevated ALT was defined as ALT ≥ 40 IU/l [16]. HBsAg was determined by an enzyme linked immuno-sorbent assay (Enzygnost, Dade Behring Marburg GmbH, Germany). Anti-HCV was determined by an EIA test (Abbott HCV EIA, third generation).

**Table 1:** – Characteristics of the study population (mean ± SD).

Variable	Men (n = 3103)	Women (n = 2447)	***P*** value
Age (yr)	49.0 ± 11.9	50.0 ± 12.8	.005
BMI (kg/m^2^)	24.6 ± 3.3	23.5 ± 3.8	
WC (cm)	87.6 ± 8.7	80.7 ± 10.2	< .0001
SBP (mmHg)	125.5 ± 16.4	118.7 ± 19.8	< .0001
DB (mmHg)	80.1 ± 10.5	74.9 ± 10.8	< .0001
ALT (IU/l)	35.5 ± 44.5	24.0 ± 21.5	< .0001
Fasting glucose (mg/dl)	104.0 ± 34.2	100.5 ± 29.3	.0001
TC (mg/dl)	201.8 ± 39.4	199.1 ± 39.3	.01
TG (mg/dl)	134.4 ± 136.2	97.6 ± 73.1	< .0001
LDL (mg/dl)	129.1 ± 34.7	122.8 ± 35.0	< .0001
HDL (mg/dl)	43.4 ± 11.0	52.4 ± 13.2	< .0001
Uric acid (mg/dl)	6.7 ± 1.4	5.4 ± 1.3	< .0001
Elevated ALT (%)	721 (23.2)	229 (9.4)	< .0001
HBsAg (+) (%)	538 (17.3)	286 (11.7)	< .0001
Anti-HCV (+) (%)	138 (4.4)	138 (5.6)	.042
Fatty liver (%)	1639 (52.8)	897 (36.7)	< .0001
Gall stone (%)	158 (5.1)	138 (5.6)	.367
Cirrhosis (%)	19 (0.6)	7 (0.3)	.077
Hepatocellular carcinoma (%)	7 (0.2)	1 (0.004)	.072
Smoker (%)	1280 (41.3)	146 (6.0)	< .0001
Drinker (%)	536 (17.3)	70 (2.9)	< .0001

Statistical analysis was performed by SPSS (Chinese Version 10.0, Sinter Information Corp, Taiwan). The *t* test distinguished the differences in the variables between men and women. The *Chi*-square test distinguished the association between the related factors and elevated ALT. The multivariate logistic regression further analyzed the significant factors that were identified in the *Chi*-square test. All *P* values less than .05 were considered statistically significant.

## 3. Results

### 3.1. Characteristics of the study population

Table 1 presents the relationship of characteristics in the study’s population. There were 3103 men (55.9%) and 2447 women (44.1%). The mean age for all subjects was 49.4 ± 12.3 years (age range of 20-87). The mean value of ALT was 35.5 ± 44.5 IU/l in men and 24.0 ± 21.5 IU/l in women (*P* < .0001). The overall prevalence of elevated ALT was 17.1%, with a significant gender difference (23.2% in men *vs.* 9.4% in women, *P* < .0001). Men had higher levels of WC, blood pressure, fasting glucose, TC, TG, LDL, and uric acid than women did. The prevalence of HBs Ag positive was 17.3% in men, and 11.7% in women (*P* < .0001). The prevalence of anti-HCV positive was 4.4% in men, and 5.6% in women. In addition, the prevalence of a fatty liver was 52.8% in men and 36.7% in women. The prevalence of cigarette smoking and alcohol drinking were 41.3% and 17.3% in men, respectively. 6% of women were cigarette smokers, and 2.9% were alcohol drinkers. Thus, there were significant gender differences in both smoking and drinking.

**Table 2:** Elevated ALT and its related factors *via Chi*-square test.

Variable	Men (n = 3103)			Women (n = 2447)		
	Normal ALT (%)	Elevated ALT (%)	*P* value	Normal ALT (%)	Elevated ALT (%)	*P* value
Age(years) (mean ± SD)	49.7 ± 12.2	46.7 ± 10.8	< .0001	49.5 ± 12.9	54.1 ± 11.3	< .0001
BMI ≥ 27			< .0001			< .0001
No	1999 (80.8)	474 (19.2)		1893 (92.0)	165 (8.0)	
Yes	383 (60.8)	247 (39.2)		325 (83.5)	64 (16.5)	
WC ≥ 80 cm (men ≥ 90 cm)			< .0001			< .0001
No	1531 (82.7)	320 (17.3)		1155 (94.4)	69 (5.6)	
Yes	851 (68.0)	401 (32.0)		1063 (86.9)	160 (13.1)	
Hypertension			.008			< .0001
No	1698 (78.1)	477 (21.9)		1736 (91.9)	152 (8.1)	
Yes	684 (73.7)	244 (26.3)		482 (86.2)	77 (13.8)	
DM			.113			< .0001
No	2098 (77.2)	619 (22.8)		2041 (91.4)	191 (8.6)	
Yes	284 (73.6)	102 (26.4)		177 (82.3)	38 (17.7)	
Hypercholesterolemia			.002			.948
No	1247 (79.1)	330 (20.9)		1177 (90.7)	121 (9.3)	
Yes	1135 (74.4)	391 (25.6)		1041 (90.6)	108 (9.4)	
Hypertriglyceridemia			< .0001			< .0001
No	1766 (80.2)	435 (19.8)		1934 (92.0)	168 (8.0)	
Yes	616 (68.3)	286 (31.7)		284 (82.3)	61 (17.7)	
Elevated LDL*			.293			.674
No	1281 (77.6)	369 (22.4)		1383 (90.9)	139 (9.1)	
Yes	1098 (76.0)	346 (24.0)		834 (90.4)	89 (9.6)	
Reduced HDL			< .0001			< .0001
No	1393 (79.8)	353 (20.2)		1201 (93.8)	80 (6.2)	
Yes	989 (72.9)	368 (27.1)		1017 (87.2)	149 (12.8)	
Hyperuricemia			< .0001			< .0001
No	1487 (79.9)	375 (20.1)		1843 (92.0)	161 (8.0)	
Yes	895 (72.1)	346 (27.9)		375 (84.7)	68 (15.3)	
HBsAg			< .0001			.075
Negative	2030 (79.1)	535 (20.9)		1967 (91.0)	194 (9.0)	
Positive	352 (65.4)	186 (34.6)		251 (87.8)	35 (12.2)	
Anti-HCV			< .0001			< .0001
Negative	2323 (78.3)	642 (21.7)		2130 (92.2)	179 (7.8)	
Positive	59 (42.8)	79 (57.2)		88 (63.8)	50 (36.2)	
Fatty liver			< .0001			< .0001
No	1243 (84.9)	221 (15.1)		1441 (93.0)	109 (7.0)	
Yes	1139 (69.5)	500 (30.5)		777 (86.6)	120 (13.4)	
Gall stone			.307			.033
No	2266 (76.9)	679 (23.1)		2100 (90.9)	209 (9.1)	
Yes	116 (73.4)	42 (26.6)		118 (85.5)	20 (14.5)	
Cirrhosis			< .0001			< .0001
No	2374 (77.0)	710 (23.0)		2215 (90.8)	225 (9.2)	
Yes	8 (42.1)	11 (57.9)		3 (42.9)	4 (57.1)	
Hepatocellular carcinoma			.003			.748
No	2380 (76.9)	716 (23.1)		2217 (90.6)	229 (9.4)	
Yes	2 (28.6)	5 (71.4)		1 (100.0)	0 (0)	
Smoker			.051			.097
No	1422 (78.0)	401 (22.0)		2080 (90.4)	221 (9.6)	
Yes	960 (75.0)	320 (25.0)		138 (94.5)	8 (5.5)	
Drinker			.001			.058
No	2000 (77.9)	567 (22.1)		2150 (90.5)	227 (9.5)	
Yes	382 (71.3)	154 (28.7)		68 (97.1)	2 (2.9)	

*Imprecise summation of total subjects was due to missing data.

**Table 3:** Related factors for elevated ALT *via* multivariate logistic regression in men.

**Variable**	**EP (SE)**	**OR**	**95% CI**
Intercept	-1.05 ( .22)		
Age (years)	- .03 ( .00)	.97	.96-0.98****
BMI ≥ 27 (yes *vs.* no)	.39 ( .13)	1.48	1.16-1.90**
WC ≥ 90 cm (yes *vs.* no)	.39 ( .12)	1.47	1.17-1.85***
Hypertension (yes *vs.* no)	.14 ( .10)	1.15	.95-1.40
Hypercholesterolemia (yes *vs.* no)	.28 ( .10)	1.33	1.10-1.61**
Hypertriglyceridemia (yes *vs.* no)	.28 ( .11)	1.32	1.07-1.63**
Reduced HDL (yes *vs.* no)	.03 ( .10)	1.03	.85-1.25
Hyperuricemia (yes *vs.* no)	.19 ( .10)	1.21	1.01-1.46*
HBsAg positive (yes *vs.* no)	.83 ( .11)	2.30	1.85-2.88****
Anti-HCV positive (yes *vs.* no)	2.10 ( .20)	8.17	5.52-12.09****
Fatty liver (yes *vs.* no)	.73 ( .11)	2.07	1.68-2.56****
Cirrhosis (yes *vs.* no)	1.46 ( .51)	4.30	1.58-11.73**
Hepatocellular carcinoma (yes *vs.* no)	1.85 ( .96)	6.33	.96-41.63
Drinker (yes *vs.* no)	.25 ( .12)	1.28	1.02-1.62*

EP: Estimated parameter; SE: Standard error; OR: Odds ratio; CI: Confidence interval.

*P < .05; **P < .01; ***P < .001; ****P < .0001

**Table 4:** Related factors for elevated ALT *via* multivariate logistic regression in women.

Variable	EP (SE)	OR	95% CI
Intercept	-3.36 ( .35)		
Age (years)	.00 ( .01)	1.00	.99-1.01
BMI ≥ 27 (yes *vs.* no)	.31 ( .19)	1.36	.94-1.98
WC ≥ 80 cm (yes *vs.* no)	.40 ( .18)	1.49	1.04-2.14*
Hypertension (yes *vs.* no)	.10 ( .18)	1.10	.78-1.56
DM (yes *vs.* no)	.31 ( .22)	1.37	.89-2.10
Hypertriglyceridemia (yes *vs.* no)	.38 ( .19)	1.46	1.01-2.12*
Reduced HDL (yes *vs.* no)	.44 ( .16)	1.55	1.14-2.13**
Hyperuricemia (yes *vs.* no)	.26 ( .18)	1.30	.92-1.83
Anti-HCV positive (yes *vs.* no)	1.90 ( .21)	6.69	4.42-10.14***
Fatty liver (yes *vs.* no)	.31 ( .17)	1.36	.98-1.88
Gall stone (yes *vs.* no)	.23 ( .27)	1.25	.74-2.13
Cirrhosis (yes *vs.* no)	1.24 ( .89)	3.47	.60-19.92

EP: Estimated parameter; SE: Standard error; OR: Odds ratio; CI: Confidence interval.

*P < .05; ** *P* < .01; ***P < .0001

### 3.2. Factors related to elevated ALT *via Chi*-square test

The results of the *Chi*-square analysis for elevated ALT and its related factors are shown in Table 2. The significant related factors of elevated ALT in men were age, obesity, hypertension, hypercholesterolemia, hypertriglyceridemia, reduced HDL, hyperuricemia, HBs Ag positive, anti-HCV positive, fatty liver, liver cirrhosis, hepatoma, and drinking. In women, the significant related factors of elevated ALT in men were age, obesity, hypertension,DM, hypertriglyceridemia, reduced HDL, hyperuricemia, anti-HCV positive, fatty liver, gallbladder stone and liver cirrhosis.

### 3.3. Related factors for elevated ALT *via* logistic regression

The related factors for elevated ALT found by multivariate logistic regression are shown in Table 3 and Table 4. Obesity, hypercholesterolemia, hypertriglyceridemia, hyperuricemia, HBsAg positive, anti-HCV positive, fatty liver, cirrhosis, and drinking were significantly associated with elevated ALT in men. Anti- HCV positive was the factor found to create the highest risk for elevated ALT in men (OR = 8.17, 95% CI = 5.52-12.09). In women, central obesity, hypertriglyceridemia, reduced HDL and anti-HCV positive were significantly associated with elevated ALT. Similarly, anti-HCV positive was the factor found to create the highest risk for elevated ALT in women (OR = 6.69, 95% CI = 4.42-10.14).

## 4. Discussion

There are thousands of enzymes within hepatocytes. Some enzymes, such as transaminases, leak into the interstitial space and plasma due to a hepatocyte injury or necrosis and cause serum levels to elevate. These enzymes catalyze and transfer the amino groups of certain amino acids, and they play a very important role in glycogenesis [6, 17]. Elevated serum aminotransaminase is normal in some healthy persons. Many investigations have found that gender and ethnicity are factors associated with ALT values. For example, men and nonwhites (such as blacks and Hispanics) tend to have higher ALT than women and whites [18, 19]. Our study also found that men (23.2%) had a higher prevalence of elevated ALT than women (9.4%).

We found that anti-HCV positive was the predominant factor related to elevated ALT in both men and women. Since the discovery of HCV and the subsequent development of a diagnostic test to measure anti-HCV, the acquisition of HCV infection has been increasingly linked to the development of various liver diseases, including impaired liver function, chronic liver disease, and histologically advanced liver disease [20]. In Taiwan, the relative important of HCV infection in causing such liver diseases has been clearly defined [2, 21, 22]. The mechanism relating impaired liver function to HCV infection possibly involves viral lytic infections, which cause destruction of liver cells and liver damage. Viral replication of HCV is extremely robust. It is estimated that more than 10 trillion virion particles are produced per day, even in the chronic phase of infection [23]. Only in men was the hepatitis B virus (HBV) infection significantly associated with elevated ALT in our study. In contrast to HCV infection, the HBV replication cycle is not directly cytotoxic to cells; instead, host immune responses to viral antigens displayed on infected hepatocytes are the principal determinants of hepatocellular damage [24].

In line with the notion that alcohol intake is a cause of chronic disease [25], our results indicated that alcohol consumption was a significant correlate for elevated ALT in men. Alcohol may injure the liver through a genotoxic mechanism or by accumulation of excess fat in the liver (fatty liver) [25], leading to compromised liver function.

With the promotion of nutrition, obesity caused by over-eating has become a major problem all over the world. Epidemiological data indicate that obesity is a risk factor for diabetes mellitus, hypertension, hyperlipidemia, cardiovascular disease, and cancer [26, 27]. Clinically, abnormal liver function test results have often been found in overweight and obese people. Numerous studies have demonstrated an important relationship between body mass index and elevated ALT [9, 28, 29]. Salvaggio *et al.* found this relation was still evident after age, alcohol consumption, physical activity, and cigarette smoking had been adjusted for [30]. Obesity may present histological liver alternations, mainly steatosis [31]. Many pathological reports revealed fat droplets or steatosis occurred frequently in the liver biopsys of overweight patients with hypertransaminase [32, 33]. This helped to explain why obesity or overweight weight would cause hypertransaminase. Moreover, some clinical studies showed that if the patients reduced their weight, their liver functions would become normal [18, 34-36]. Our study also showed that people with central obesity were more likely to have elevated ALT than people without central obesity.

In addition, our study showed that hypertriglyceridemia had an effect on elevated ALT. Goncales *et al.* reported that hypertriglyceridemia and hypercholesterolemia were associated with elevated ALT [37]. But few reports have addressed the relationship between serum triglyceride level and ALT level [38, 39]. We therefore do not know the detailed mechanisms on how serum triglyceride affects ALT. This issue needs further investigation.

The major limitation of this study was that our definition of elevated ALT was based on a single measurement, and the value may fluctuate over time. Thus, this cross-sectional study may blur etiologic associations because of ‘incidence-prevalence bias [40]. Hence, a long-term prospective study is needed to confirm the findings made herein. Moreover, the limitation of this study was that the status of the hepatitis B e antigen (HBe Ag) was not measured. In HBV infections, the most likely viral antigens targeted in the immune response are hepatitis B core antigens and HBe Ag [41]. Thus, when HBV replication is sustained, as indicated by positivity for HBe Ag, continuous cycles of hepatocellular injury may occur from immune attacks.In conclusion, this retrospective study has found that central obesity, hypertriglyceridemia, and anti-HCV positive are factors predominantly related to elevated ALT in both men and women.
